# “Title does not dictate behavior”: Associations of formal, structural, and behavioral brokerage with school staff members’ professional well-being

**DOI:** 10.3389/fpsyg.2022.885616

**Published:** 2022-07-19

**Authors:** Beat Rechsteiner, Miriam Compagnoni, Katharina Maag Merki, Andrea Wullschleger

**Affiliations:** Institute of Education, Faculty of Arts and Social Sciences, University of Zurich, Zurich, Switzerland

**Keywords:** brokerage, principals, teachers, professional development, social capital, professional well-being

## Abstract

Individuals in brokerage positions are vital when further developing complex organizations with multiple subgroups only loosely coupled to each other. Network theorists have conceptualized an individual’s brokerage as the degree to which a person occupies a bridging position between disconnected others. Research outside the school context has indicated for quite some time that an individual’s social capital in the form of brokerage is positively associated with professional development—not only on a collective but also on an individual level. Schools are without any doubt complex organizations with multiple loosely connected stakeholders involved when further developing their educational practice. Thus, it is not surprising that in recent years, the concept of brokerage has gained interest in research on school improvement as well. Up to now, in school improvement research brokerage has been operationalized in different ways: as individuals’ formal entitlement to act as intermediaries (formal brokerage), their position within a social network (structural brokerage), or their behavior when linking disconnected groups of staff members (behavioral brokerage). As these perspectives have often been examined separately, this study, as a first step, aimed to simultaneously assess school staff members’ formal, structural, and behavioral brokerage, and examine their degree of interrelatedness. In a second step, associations of brokerage with professional well-being were analyzed. Even though there is evidence for the positive impact of brokerage on professional development, only little is known about its associations with professional well-being. In a third step, interaction effects were examined when formal brokerage is congruent or incongruent with other facets of brokerage. Based on a sample of 1,316 school staff members at 51 primary schools in the German-speaking part of Switzerland, we conducted both bivariate correlational and multiple-group structural equation modeling analyses. The findings revealed that formal, structural, and behavioral brokerage are interrelated facets. However, formal entitlement did not determine either structural position or behavior. Moreover, brokerage within schools was only partially related to professional well-being. In the discussion section, the study’s key contributions and practical implications are presented in detail.

## Introduction

Previous research outside of educational science has indicated that an individual’s social capital in the form of brokerage is related to professional development—both collectively and individually (i.e., Burt, 2005; Ward et al., 2009; Meyer, 2010; Stovel and Shaw, 2012; Long et al., 2013; Obstfeld et al., 2014; Kwon et al., 2020). Brokerage can be conceptualized as the complex processes when individuals occupy a bridging position between disconnected others and facilitate interactions among them (Halevy et al., 2019). This bridge-building may function as an “engine of endogenous change” (Burt, 2005), as being in an intermediary position allows brokers to gain a different perspective on their own everyday practice by seeing what others do, learning what others know (or don’t know), and realizing what others struggle with. In this way, their everyday practice may appear in a new light, making their own strengths and weaknesses more visible. This may then function as a stimulus for further developing and adjusting their own practice and routines.

In the last decade, a growing number of studies in research on school improvement have focused explicitly on brokerage (i.e., Spillane and Kim, 2012; Daly et al., 2014b; Neal et al., 2019; Van Gasse et al., 2019; van den Boom-Muilenburg et al., 2022). This is not surprising, as there is a long tradition of theoretically framing educational systems as complex landscapes in which various actors within, between, and beyond schools constantly work the interface between loosely coupled subsystems (Weick, 1976; Shen et al., 2017)—such as different classrooms and departments within a school, different schools within a local school district, or school districts within a regional or national school system. The overarching aim in studies on brokerage in the domain of school improvement is to better understand both formal and informal activities when bringing disconnected groups within the school context together. This may be in terms of how knowledge is transferred from research to practice (Cooper, 2012; Brown, 2019; Neal et al., 2019), from school district to school (Park and Datnow, 2009; Daly et al., 2014a,b), from school to school (Kolleck, 2016), or within schools (Slavit and Roth McDuffie, 2013; Jusinski, 2019; van den Boom-Muilenburg et al., 2022).

In this current study, we narrowed our focus on the loose coupling of different departments and subgroups within schools. Previous research indicated that schools are often organized in multiple departments and subgroups not necessarily aligned in terms of the direction they take when further developing their educational practice and often function autonomously rather than in an orchestrated way (Feldhoff et al., 2010; Farley-Ripple and Grajeda, 2019). We argue that the brokerage concept is essential to analyze how disconnected subgroups within a school may be linked when striving for effective collaboration to change educational practice (i.e., Carmichael et al., 2006; Spillane and Kim, 2012; Nordholm, 2016).

However, there are still multiple shortcomings of the brokerage concept when it comes to school improvement research and in particular concerning school staff members as bridge-builders within their schools’ professional networks.

*First*, brokerage is a multifaceted phenomenon rather than a unidimensional concept (Long et al., 2013). Hence, brokerage has been examined not from a single but from multiple angles (Stovel and Shaw, 2012; Kwon et al., 2020). In previous research, formal, structural, and behavioral perspectives to assess an individual’s brokerage can be differentiated. These perspectives all share commonalities conceptually but stem from different research traditions—for instance, in terms of the underlying theoretical assumptions or the methodological approaches to identifying brokerage. However, up to now these perspectives have often been examined separately and the relationships between these different aspects of brokerage have not been considered sufficiently in empirical research on school improvement. It is therefore not clear to what degree these different aspects of brokerage deliver information that may be used either interchangeably or as complementary. Accordingly, the first aim of this study was to examine to what degree individual teachers’ titles (being appointed to act as a formal teacher leader) are related to their structural positions (bridging structural holes in a network), and whether the formal title or structural position dictates teachers’ network behavior (acting as bridge-builders between different groups of actors).

*Second*, whereas there is ample evidence that being in a brokerage position brings advantages for professional development, there is only little research on the associations of brokerage with professional well-being. However, a high level of professional well-being has proven to be of crucial importance when changing a school’s educational practice (Creemers and Reezigt, 1996). The few studies beyond the school context that examined these relationships are rather inconsistent and highly dependent on the aspect of an individual’s professional well-being that was assessed. Burt (1992, 2005), for instance, proclaimed that brokers not only do better in terms of professional development but also feel more powerful and successful when negotiating agreements and experience themselves as more competent professionally. Other researchers found that being in between different groups may also have harmful consequences on an individual’s psychological well-being (i.e., Krackhardt, 1999; Wenger, 1999; Carboni and Gilman, 2012; Mollenhorst et al., 2015). The argumentation is that being simultaneously part of multiple groups is often stressful and full of challenges. However, the associations of brokerage and professional well-being have not yet been studied thoroughly in the school context. This is a major concern, as brokerage is highly context-specific (Kwon et al., 2020). Building bridges among disconnected others in schools, and in particular school staff members the closest to the classroom, such as teachers and principals, differ from other organizations and their employees—for instance, in terms of organizational (leadership) structures, career paths, and available incentives to foster more organizational commitment (Lortie, 1975; Ingersoll and Collins, 2018). Hence, the second aim of this study was to explore the relationships between school staff members in a brokerage position and their professional well-being in a multifaceted way. To this end, professional well-being was conceptualized by three overlapping themes that represent both *hedonic* and *eudaimonic* approaches to assessing an individual’s professional well-being (Deci and Ryan, 2008): work-related stress, the experience of competency in further developing educational practice, and job satisfaction. Whereas a low level of work-related stress represents the absence of negative affect (*hedonic well-being*), having a meaningful professional life (*eudaimonic well-being*) was assessed through an individual’s experience of competency in further developing the school’s educational practice. Finally, the third theme, an individual’s job satisfaction, overlaps these two traditions, as it stands for a more general evaluation of individuals’ professional well-being combining both the absence of negative affect and having a meaningful job.

*Third*, some studies in school improvement research found that a lack of legitimacy (e.g., not having a formalized brokerage position) is a constraining factor in the benefits of brokerage—both on an individual and collective level (Hopkins et al., 2013; Nordholm, 2016). It therefore seems vital to formalize school actors’ brokerage, for instance in the form of middle or teacher leadership groups (Grootenboer et al., 2019), to leverage the potential of brokerage when further developing educational practice (Spillane et al., 2018). However, another study in the domain of school improvement warned about formalizing brokerage by implementing new hierarchical structures (Jusinski, 2019). These structures may threaten teachers’ “autonomy parity pattern” (Lortie, 1975)—whereby teachers want autonomy for their work, do not accept interference by outsiders, and demand equal treatment of all teachers (Rowan, 1994; Feldhoff et al., 2010). As a consequence, by formalizing school actors’ brokerage their credibility among their peers may decline (Jusinski, 2019). To analyze this inconsistency in previous research, we examine an interactional effect when formal brokerage is congruent or incongruent with other facets of brokerage on the outcome variables of professional well-being.

Bringing these three concerns together, the overarching aims of this article are to analyze brokerage and professional well-being in a more comprehensive way and to investigate the relation of brokerage to school staff members’ professional well-being. To this end, we examined primary schools in the German-speaking part of Switzerland, where school staff members often organize in subject-related departments or *Zyklusteams* (which can be translated as a team of teachers based on the students’ age group, such as lower and upper primary classes) (Eurydice, 2022).

In the following sections, we first provide a more in-depth picture of the multifaceted concepts of brokerage and professional well-being. We then theoretically frame and empirically report how the two concepts of brokerage and professional well-being may correspond with each other. Based on that, we then outline our research questions and hypotheses.

### Brokerage: A multifaceted concept

Hargreaves and Fullan (2015) indicated that school staff members’ professional development is influenced both by an individual’s human and social capital. Whereas human capital can be conceptualized as individuals’ pedagogical and work-related knowledge and their strategies how to increase this knowledge (Mitchell and Sackney, 2011) a school staff member’s social capital is about their social relationships within and beyond their school (Hargreaves and Fullan, 2015). In previous research social capital has been conceptualized and used in different ways. It is beyond the scope of this publication to give an overview of all these different conceptualizations. However, Coppe et al. (2022) provide a critical reflection in this special issue on the use of social capital in teacher research both in terms of theoretical assumptions and methodological approaches. They pointed out that studies using social capital as a theoretical lens need to clearly define what kind of social capital they are referring to—for instance, whether the focus is on an individual or collective level. In this study, we refer to the work of Crossley et al. (2015) to conceptualize an individual’s social capital as brokerage. Crossley et al. (2015) distinguished three different versions of social capital: “one focused upon access to resources; one focused upon social cohesion; and one focused upon ‘brokerage’ across ‘structural holes”’ (p. 26). In contrast to social capital as resources or cohesion the brokerage version of social capital has an explicit focus on change processes and how individuals may foster or constrain professional development (for an overview see Crossley et al., 2015). Previous scholars defined social capital in the form of brokerage as the degree to which individuals act as in-betweens that facilitate “transactions between other actors lacking access or trust in one another” (Marsden, 1982). The definition of brokerage is widely accepted, but the concept has been analyzed from different angles—from a formal, structural, and behavioral perspective.

*From a formal perspective*, principals and teacher leaders may act as brokers by coordinating the flow of information across different subgroups within an organization (Feldhoff et al., 2010; Wenner and Campbell, 2017). Following the formal approach to brokerage by Gould and Fernandez (1989), school staff members in a brokerage position may function as *gatekeepers* and *representatives* at the same time. As gatekeepers they filter information from outside to their inner circle. As representatives they inform others what issues a specific group is dealing with at the moment. Hence, they can be viewed as bottlenecks that have the mandate to manage both top-down (i.e., school to an individual staff member) and bottom-up (i.e., individual or sub-team to school) change processes.

In the context of school organizations and their development, *steering groups* are an example of and a particular kind of a formal leadership group that is in a brokerage position (Feldhoff et al., 2010; Tulowitzki et al., 2021). Steering groups are a composite of the school’s principal and teacher leaders. The steering group members function as intermediaries between school staff and the school’s leadership group. In a best-case scenario, they act as bridge-builders that balance the needs and interests of the subgroup they are representing and the needs and interests of the school’s leadership group. In their brokerage position they may act as formal gatekeepers, supporting the implementation of school-wide change processes from the center to the utmost periphery of the organization. In reality, however, steering groups are also an “area of tension” (Feldhoff et al., 2010). This is due to the fact that a shift in school staff members’ identity from *primus inter pares* [first among equals] to a more managerial role can lead to a fundamental reinterpretation of school leadership, internal hierarchies, and professional roles (Peetz, 2015) as well as to a more aligned educational practice (Scheerens, 2012). For instance, teachers may worry that more collective sensemaking processes to define common goals of school improvement and negotiating a consensus threatens their own agenda or vision of how to improve educational practice (Vangrieken et al., 2017). Thus, some school actors may see the implementation of such new cooperative structures as a loss of their autonomy (Vangrieken and Kyndt, 2020). Therefore, a teacher being formally appointed (for instance by the principal) to act as a broker between internal subgroups and the school’s leadership group may be more or less accepted by colleagues (Hopkins et al., 2013; Nordholm, 2016), which may lead to more stress for the teacher and reduced perceived benefit of the brokerage position. As a consequence, school staff members may reject or look critically at these formal middle management structures.

*From a structural perspective*, brokerage is analyzed in terms of network structures. Brokers differ from other actors in a social network by bridging structural holes between disconnected individuals; they therefore have opportunities to link people but also to control the flow of information (Burt, 1992; Wasserman and Faust, 1994). A structural hole occurs in a social network when two actors are not directly connected. According to Burt (2005), an individual’s social capital is a function of brokerage across these structural holes. Hence, social capital is determined not only by formal roles but also by informal ties between actors. It is therefore vital to analyze structural brokerage based on social network analysis when examining dynamics in both formal and informal cooperative structures (Burt, 2005). The most straightforward way to measure an individual’s degree of being in a brokerage position is the betweenness-centrality (Freeman, 1977). This coefficient is a standardized number of paths going through an individual actor when connecting every other actor in the network on its shortest path. Within a school’s social network, an individual with a high betweenness-centrality is perceived by others to be located in-between different subgroups of staff members and this way potentially acts as knowledge manager, capacity builder, and linking agent within the web of a school’s actors (Ward et al., 2009).

Up to now, only a few studies have focused on school staff members in structural brokerage positions. However, Daly and colleagues examined cooperative practice among educational leaders on a school and system level (Daly et al., 2014a,b). They indicated that although district leaders are well-positioned in terms of their formal entitlements to act as brokers, they often do not exploit their potentials. Additionally, they pointed out that it is foremost principals as informal and formal brokers, identified based on social network data, who play a vital role in school-to-school cooperation. Further, Hopkins et al. (2013) examined structural brokerage to compare formal and informal network structures when redesigning educational infrastructure on a district level. They found that teacher leaders emerged as essential brokers that help to craft coherence in visions and goals of educational change across different groups of actors within and outside of school. Moreover, in a mixed-method study examining school actors’ structural brokerage, Spillane and Kim (2012) found that formal organizational structures have a substantial influence on informal network structures. They pointed out that school organizations embracing a more distributed leadership approach enabled highly credible staff members to act as brokers from a legitimate position. All of these studies put an emphasis on analyzing dynamics between formal and structural brokerage. They all agreed that formal entitlement does not determine but rather influences an individual’s structural network position.

However, Obstfeld et al. (2014) argued, *from a behavioral perspective*, that simply being in a brokerage position does not necessarily lead to actual brokerage behavior. Rather than treating social network structure as a determinant of whether an actor is able to act as a broker, they argued that although network structures affect the ways a broker acts, they do not define brokerage behavior. They conceptualized brokerage as a “behavior by which an actor influences, manages, or facilitates interactions between other actors” (Obstfeld et al., 2014). They pointed out that brokerage behavior may appear in different forms: as a *conduit*, *tertius gaudens*, or *tertius iungens*. Whereas *conduits* (“the third who transfers”) facilitate knowledge transfer by acting as neutral messengers, brokers with a *tertius gaudens* orientation (“the third who enjoys”) take advantage of their prominent position, for instance by cultivating conflict between individuals or keeping other actors separate. In this way, brokers make sure that they stay in their exclusive and powerful positions, transferring, hoarding, or even manipulating information as it pleases their situation. Previous studies discussed these power dynamics and problematic aspects of brokerage activity (McGrath and Krackhardt, 2003; Burt, 2005; Kislov et al., 2017). In contrast, brokers with a *tertius iungens* orientation (“the third who joins”) foster collaboration among others, by introducing different persons to each other and coordinating new collaborative action. Obstfeld et al. (2014) pointed out that organizational change benefits the most when central actors show a *tertius iungens* orientation. Moreover, in this study we refer to school staff members as crucial agents of change actively shaping their professional networks in such a way that collaboration among all staff members is increased and the school’s educational practice is improved both sustainably and collectively (Mitchell and Sackney, 2011). Therefore, in the following we refer to the *tertius iungens* orientation only when using the term brokerage behavior.

The behavioral brokerage perspective has so far been rather neglected when it comes to research on school staff members. Moolenaar et al. (2014), as an exception, revealed that school staff members “who are more intentional about brokering connections between others, also tend to share new ideas with more others and in turn perceive their school’s climate to be more innovative” (p. 116). Moreover, a study examining teacher involvement in school improvement based on brokerage behavior (Rechsteiner et al., 2022) found that teachers’ brokerage behavior is related to their perception of the school’s leadership practice and has a positive association with their involvement in further developing the school’s educational practice. However, little is known up to now on how school staff members’ behavioral brokerage corresponds with the other facets of brokerage outlined above.

Moreover, previous research has neglected to analyze the relation between the formal, structural, and behavioral facets of brokerage. Different combinations of these facets are imaginable, as in the following examples:

• Someone is formally appointed to act as a broker, is actually seen by colleagues as a broker, and actively applies brokerage strategies.
• Someone is only formally appointed, is not accepted by others, and does not show any brokerage behavior.
• Someone acts as a broker, is structurally visible in the school’s professional network, but is not formally appointed to act as a broker.

In terms of these multiple facets of brokerage, in this study our research questions address to what degree formal, structural, and behavioral brokerage correspond, and whether they function as complements.

In sum, based on previous research we assume that the different facets of brokerage cannot be used interchangeably (i.e., Obstfeld, 2005; Daly et al., 2014a,b). However, we further argue that formal entitlement, structural position, and bridge-building behavior are interrelated and function as complements. In the following section we elaborate on the intertwined nature of these three facets when focusing on brokerage and its relation to professional well-being.

### School staff members’ professional well-being

Previous research highlighted the importance of school staff members’ professional well-being for individual and collective professional development (i.e., Hascher, 2010; Bermejo-Toro et al., 2016; Turner and Thielking, 2019; Hascher and Waber, 2021; Pöysä et al., 2022). School staff members that feel well at work are more committed to taking an active part in school improvement (Creemers and Reezigt, 1996). Moreover, in a systematic review on teacher well-being, Hascher and Waber (2021) pointed out that teachers reporting high professional well-being show better instructional quality.

As there is no single definition and conceptualization of well-being, Linton et al. (2016) suggested using the concept as an umbrella term. In this study we also conceptualized school staff members’ professional well-being “as a multidimensional construct, reflecting themes that often overlap” (Linton et al., 2016; p. 13). This study addressed the overlapping themes of work-related stress, the experience of competency when further developing educational practice, and job satisfaction. These themes refer to Deci and Ryan’s (2008) work on analyzing well-being from both a hedonic and eudaimonic tradition. Following the hedonic approach, most prominently represented by the work of Diener (1984, 2009), professional well-being can be conceptualized as the absence of negative affect. In this study, therefore, hedonic professional well-being was assessed in terms of school staff members’ work-related stress level. Thus, a high level of professional well-being means that individuals reported a low level of stress. However, experiencing work-related stress does not necessarily mean that individual school staff members are not happy with their job. Thus, from an eudaimonic perspective, professional well-being is about having a meaningful job and thus experiencing a high level of competency at the workplace (Waterman, 1993). In this study, therefore, we looked at an individual’s experience of competency regarding further developing educational practice in particular. We are aware that these two facets of professional well-being are interdependent dimensions. To emphasis this overlap between a hedonic and eudaimonic professional well-being we therefore, as a last theme, also assessed an individual’s job satisfaction. We argue that satisfaction with the job may be an expression of both hedonic or eudaimonic professional well-being and therefore functions as a link between these two traditions.

But how does professional well-being relate to school staff members’ brokerage? Previous research found that teachers’ social relationships play a major role if someone is feeling well or not (Hascher and Waber, 2021). Thus, when implementing new organizational structures to increase school staff members’ involvement in school improvement, it is crucial to understand how these structures may impact school staff members’ professional well-being. Or in other words, when brokers within schools feel significantly worse than their colleagues, caution is advised when formalizing bridge-building structures (i.e., by implementing steering groups). In the following section we therefore provide an overview of how brokerage and professional well-being are interrelated concepts.

### Brokerage and professional well-being

There are several reasons why brokerage is said to be related to individual and collective professional development. First, by mobilizing knowledge across groups of actors, brokers have immediate access to non-redundant information and innovative ideas. Second, they may filter, distort, or hoard resources available only to them by controlling the flow of information. Third, they may disrupt (dysfunctional) routines, as they introduce new perspectives on daily practices (McGrath and Krackhardt, 2003; Burt, 2005; Obstfeld, 2005; Lomas, 2007; Ward et al., 2009; Meyer, 2010). In this way, brokers generate innovative ideas, increase the quality of creative work, make advice and knowledge more accessible, and can act synergistically with network cohesion and strong ties to produce environments in which collaboration can flourish (Long et al., 2013; Kwon et al., 2020). Although there is ample evidence for positive relationships between brokerage and professional development, research on the associations of brokerage with professional well-being is scarce. However, we argue that the findings on professional development listed above allow us to formulate hypotheses in an exploratory way on how brokerage relates to professional well-being.

*Brokerage and work-related stress (hedonic professional well-being).* On the dark side of brokerage, previous studies indicated that being in-between different groups of actors may result in higher stress (Krackhardt, 1999; Kislov et al., 2017). Krackhardt (1999) pointed out that especially brokers having strong ties to the various groups they are connecting may experience stress, as they are more often confronted with incompatible expectations on either side, need to balance the interests of different groups, and risk violating norms that are crucial in one but not the other group. Therefore, Krackhardt (1999) referred to brokerage also as “the ties that torture.” In research beyond the professional context with a focus on friendship networks, previous research also found negative associations between brokerage and psychological well-being (Carboni and Gilman, 2012; Mollenhorst et al., 2015). Up to now, only one qualitative study (Jusinski, 2019) analyzed the relation between brokerage and stress. Jusinski found that educators in an intermediary position more often risked overload and burn-out, as they were vulnerable to exploitation on both sides of the gaps that they were bridging. Moreover, the fine-grained approach of this present study in assessing multiple facets of brokerage simultaneously allows to test whether this first assumption holds to be true for formal, structural, and behavioral brokerage.

*Brokerage and experience of competency when further developing educational practice (eudaimonic professional well-being).* To our knowledge, there is no empirical study available that examined the associations of brokerage with an individual’s experience of competency. However, following Burt’s (2005) assumption that individuals in a brokerage position not only do better but also feel better, there are theoretical arguments that support the claim that brokers feel more competent when further developing educational practice compared to their colleagues. Burt argued that brokers are less constrained in their professional networks and therefore have more leeway to shape their social worlds (Burt, 1992, 2005). As a consequence, they experience themselves as more powerful and have more success when negotiating agreements (i.e., Stovel and Shaw, 2012; Quintane and Carnabuci, 2016). Regarding the school context, previous research found that being in a brokerage position is positively associated with a higher level of self-confidence and the experience of more autonomy at work (Hopkins et al., 2013; Slavit and Roth McDuffie, 2013). We therefore assume that teachers and principals in brokerage positions also tend to feel more competent when it comes to changing the school’s educational practice. Again, in the current study this claim was examined in a differentiated way by analyzing it regarding the three different facets of brokerage.

*Brokerage and job satisfaction (combining hedonic and eudaimonic professional well-being)*. It is quite likely that individuals’ having access to different social worlds and being in an exclusive and powerful position to control the flow of information within a professional network are more satisfied than others with their jobs. Moreover, having more success in terms of professional development may also result in higher job satisfaction. However, it is also reasonable to assume that brokerage is negatively correlated with job satisfaction. For example, in Wenger’s (1999) communities of practice theoretical framework, Wenger pointed out that brokers more likely experience isolation by only connecting but not really belonging to any group. The feeling of being isolated within a team may have a negative impact on an individual’s job satisfaction. If school staff members’ brokerage was indeed associated with a higher job satisfaction was tested regarding their formal role (being a member of the school’s steering group), their structural network position (bridging structural holes in the school’s professional network), and their network behavior (applying brokerage activities).

*Differential effects of brokerage on professional well-being.* We assume that having or not having the formal mandate to act as a broker may moderate the relationship between brokerage and professional well-being. This is due to the fact that previous studies yield an inconsistent picture when it comes to the dynamics between formal and both structural and behavioral brokerage. Whereas some studies found that informal leaders within a team lack legitimacy to be brokers (Hopkins et al., 2013; Nordholm, 2016), others indicated that by formalizing educators as brokers, credibility among teacher colleagues declines, and the effectiveness of brokerage activity is diminished (Jusinski, 2019). In the following, we therefore analyze whether formal brokerage moderates the relationships between other facets of brokerage (structural and behavioral) and a school actor’s professional well-being. Following Nordholm’s (2016) argumentation, it may be the lack of formal legitimacy that constrains the positive impact of brokerage on well-being. We therefore assume that congruence between school staff members’ formal mandate to act as a broker and both their structural position and bridge-building behavior is more significantly associated with an individual’s professional well-being.

In sum, we argue that the relationships between brokerage and professional well-being still need to be validated, also in larger samples. In particular, there is no clear picture of whether the empirical evidence on the relations between brokerage and professional well-being from research outside of the school context is transferable to school staff members in intermediary positions within schools. This transfer is not trivial, as school staff members work, for instance, in a less hierarchical working environment than that found in in private companies or in public organizations in the health sector, where most of the studies mentioned above were conducted.

### Research questions and hypotheses

Bringing these theoretical assumptions and empirical evidence together, the aims of this study are to examine different aspects of brokerage and analyze how social capital in the form of brokerage is associated with school staff members’ professional well-being.

Our *first research question* is: To what extent do the different aspects of brokerage (formal, structural, and behavioral) interrelate?

• *Hypothesis 1a:* Formal brokerage is positively associated with school staff members’ structural brokerage position in the school’s professional network.
• *Hypothesis 1b:* Formal brokerage is positively associated with an individuals’ brokerage behavior.
• *Hypothesis 1c:* Structural brokerage is positively related to school staff members’ brokerage behavior.

Our *second research question* is: To what extent do the different brokerage aspects correspond to professional well-being?

• *Hypothesis 2a:* Brokerage is related to more work-related stress.
• *Hypothesis 2b*: Brokers experience themselves as more competent when further developing educational practice.
• *Hypothesis 2c:* Brokers tend to be more satisfied with their jobs.

Our *third research question* addresses the issue of whether being formally appointed to act as a broker moderates the relationships between structural and behavioral brokerage and professional well-being.

• *Hypothesis 3a:* Congruence in formal and other facets of brokerage is related to a lower work-related stress level.
• *Hypothesis 3b:* Congruence in formal and other facets of brokerage is associated with a more positive experience of competency.
• *Hypothesis 3c*: Congruence in formal and other facets of brokerage is related to a higher level of job satisfaction.

## Materials and methods

To answer these research questions, we relied on a complex set of multiple data sources, such as online survey, social network, and daily practice logs data. The following sections provide detailed information on: the study design and sample, the measures applied, and the data analysis.

### Study design and sample

This study was conducted in the German-speaking part of Switzerland, where over the last decades, numerous schools have adopted a formal brokerage approach by establishing steering groups to coordinate different driving forces to further develop a school’s educational practice (Dubs, 2005). Except for two schools, all schools in our sample had implemented formal steering groups. This is quite surprising, as there is no federal or cantonal legislation stipulating such formal middle management infrastructures in primary schools. But the existence of steering groups in almost all of the sampled schools indicates that this kind of formal brokerage can be considered a common (and not exceptional) practice in primary schools in the German-speaking part of Switzerland. Although steering groups are popular, however, there is only scant evidence regarding the effectiveness and differences in the local manifestations of these formal middle management structures in this context. This study does not aim to examine differences in the manifestation and quality of steering groups on a school level *per se*, but we argue that our research on brokerage, situated on an individual level, may function as a starting point for further (multilevel) analysis in this field.

As part of a larger project, data was collected from multiple school actors, such as principals, and teachers (*N*_*individuals*_ = 1,652; *N*_*schools*_ = 59). However, as not all schools participated in filling out daily practice logs, we excluded two schools from the sample. The data from three other schools were not included, as the schools’ response rates for the daily practice logs were lower than 40%. Moreover, a few schools had a response rate lower than 70% in terms of the social network data. Since the robustness of centrality measures (such as the betweenness-centrality) relies on high enough response rates (Borgatti et al., 2006) three more schools and its participants were excluded from this study. Accordingly, for this study, we focused on the responses of 1,316 principals and teachers at 51 primary schools. All of the participants took part in the study on a voluntary basis and actively gave their informed consent to participate. Data used in this study were collected both by an online questionnaire at the beginning of the school year 2019/20 (response rate on a school level (in%): *M* = 86.5, *SD* = 7.6; *Min* = 70, *Max* = 100) and daily practice logs in three different waves, each lasting a week from Monday to Sunday (response rate in terms of all the practice logs on a school level (in%): *M* = 70.0, *SD* = 10.9; *Min* = 45.6, *Max* = 95.3).

Descriptive results for the school actors showed that 87.1% were women, and the range of years of experience at the current school was 0–45, (*N* = 1,123; *M* = 9.72, *SD* = 9.03). The sampled schools differed in size (number of staff: *M* = 25.8, *SD* = 16.0; *Min* = 6; *Max* = 72), regional context (*1* = rural to *9* = urban; *Median* = 4; *Min* = 1, *Max* = 8), and socioeconomic background of the local community (welfare ratio (in%): *M* = 2.26, *SD* = 1.7; *Min* = 0.5, *Max* = 6.3; taxable income (in CHF): *M* = 33,971, *SD* = 10,924; *Min* = 16,183, *Max* = 64,735).

A possible sampling bias was analyzed by comparing teacher demographics (gender and seniority) and school characteristics (size, regional context, and socioeconomic background) with data on all Swiss primary schools provided by the Swiss Federal Statistical Office (Federal Statistical Office [FSO], 2020). As no significant differences were found, a sampling bias could be excluded. Therefore, the database proved to be a solid ground to address our research questions.

### Measures

#### Formal brokerage

Whether an individual was formally appointed to be a broker was assessed using the information provided *via* self-report in the online questionnaire on school staff members being (= 1) or not being (= 0) members of the school’s steering group or change/quality management group (*N* = 1,316*; M* = 0.14, *SD* = 0.35). There were 184 school actors formally appointed and 1,132 not formally appointed to a brokerage position.

#### Structural brokerage

The online questionnaire contained a social network question about selecting all the actors in the respondents’ school with whom they had exchanged ideas to further develop the school’s educational practice. To assess an individual’s structural brokerage, a standardized betweenness-centrality was measured (Freeman, 1977). Values between 0 and 1 indicated to what extent a school staff member is located on shortest paths when connecting all the individuals in a network (*N* = 1,129*; M* = 0.03, *SD* = 0.05, *Min* = 0, *Max* = 0.46): Higher values indicate high structural brokerage. As school size in terms of numbers of staff members influences the theoretical number of bridges an actor can possibly build, we used the standardized betweenness-centrality which takes into account the school’s size when computing a betweenness-centrality score for each individual (*N_*schools*_* = 51; *Min* = 6, *Max* = 72). Additionally, this variable was multiplied by the factor of 100 to obtain more comparable ranges of the variance for all the applied variables in our statistical model (*N* = 1,129*; M* = 2.77, *SD* = 5.08, *Min* = 0, *Max* = 45.8).

#### Behavioral brokerage

School staff members’ bridge-building behavior was assessed using a short form of Obstfeld’s “Brokerage *tertius iungens* orientation” measurement instrument (Obstfeld, 2005). In the original version, the brokerage orientation was assessed based on six items with a 7-point Likert scale (Cronbach’s alpha = 0.88). Due to the principle of parsimony, we assessed only four of these items, covering different aspects, such as whether teachers see opportunities for collaboration between people or whether they introduce school actors to each other who might have a common strategic work interest (see Table 1). The test instrument was assessed using a 6-point Likert scale ranging from 1 (*strongly disagree*) to 6 (*strongly agree*). Results revealed a high reliability of the test instrument (*N* = 1,114*; M* = 3.77, *SD* = 0.91; Cronbach’s α = 0.85*). Further*, a confirmatory factor analysis was conducted to test empirically our theoretical measurement instrument adapted from Obstfeld (2005). The measurement instrument based on four items revealed acceptable model fit indices: χ*^2^* (2) = 31.274, p < 0.001, scaling correction factor Yuan-Bentler correction (Mplus variant) = 1.456: CFI = 0.98, TLI = 0.93; RMSEA [90% CI] = 0.138 [0.098 –0.183], SRMR = 0.026. We are aware that the values for the root mean square error approximation (RMSEA) clearly deviate from the cut-off value close to.06 suggested by Hu and Bentler (1999). However, as Kenny et al. (2015) indicated, the RMSEA in properly specified models with a small number of degrees of freedom (in our case *df* = 2) often falsely indicates a poor fitting model. We therefore evaluated the goodness of fit for our latent construct based on all the other alternative fit indices not or less dependent on the degrees of freedom (such as CFI, TLI, and SRMR). As all of these values revealed an acceptable fit, we went on to address our research question based on our measurement instrument. Moreover, intraclass correlation coefficients (ICC_1_ = –0.010; ICC_2_ = –0.283) indicated that school actors did not resemble each other based on the school they belonged to Lüdtke and Trautwein (2007). Hence, behavioral brokerage, as we have theoretically outlined above, is examined most suitably on an individual level.

**TABLE 1:** Measurement instrument to assess school actors’ behavioral brokerage.

Item	N	*M*	*SD*	r*^it^*	α-drop	α
1. I introduce people to each other who might have a common strategic work interest.	1,115	3.48	1.19	0.73	0.78	–
2. I see opportunities for collaboration between people.	1,118	4.05	1.03	0.68	0.81	–
3. I point out the common ground shared by people who have different perspectives on an issue.	1,116	3.79	1.03	0.58	0.85	–
4. I introduce people when I think they might benefit from becoming acquainted.	1,115	3.77	1.16	0.75	0.77	–
Latent construct Behavioral brokerage (strongly disagree = 1; strongly agree = 6)	1,170	3.77	0.91	–	–	0.85

*M = mean and SD = standard deviation. r^it^ indicates item-total correlation coefficients. α-drop indicates Cronbach’s alpha of latent construct if item is dropped. α indicates Cronbach’s alpha of the latent construct.*

#### Professional well-being

**FIGURE 1:**
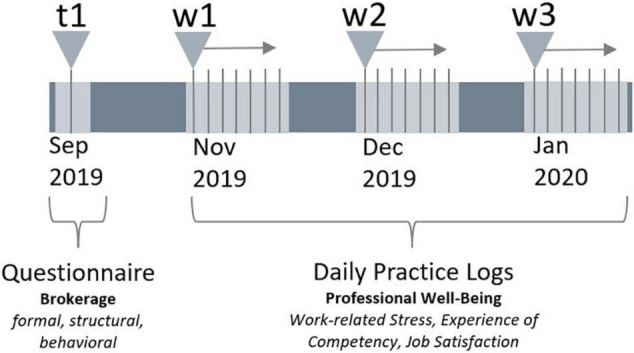
Illustration of the data collection for both the online questionnaire at the beginning of the school year 2019/20 and the three waves in which daily practice logs were collected.

In three waves, each lasting a week from Monday to Sunday, the school staff members were asked to fill out daily practice logs (see Figure 1). Each of the three aspects of school staff members’ professional well-being was assessed with a single item on a 10-point Likert scale ranging from 1 (*low*) to 10 (*high*). *Work-related stress* was measured by an item asking whether school staff members experienced their working day as stressful all in all. On average, the school staff members in our sample reported being moderately stressed (*N* = 1,150*; M* = 4.33, *SD* = 1.75). In terms of *experiencing competency* when it comes to further developing the school’s educational practice, we asked the participants when thinking back on their past working day, whether they considered it to be fruitful to further developing the school’s educational practice. The results indicated that the participants experienced themselves as neither very incompetent nor competent—although variation among the participants was high (*N* = 1,150*; M* = 5.05, *SD* = 1.96). School staff members’ *job satisfaction* was assessed by asking whether they felt satisfied with their working day all in all. In general, the participants reported being quite satisfied (*N* = 1,151*; M* = 7.86, *SD* = 1.04). The different themes of professional well-being were each aggregated separately as a mean score across the three weeks for every participant. In this way, a school staff member’s average stress level, experience of competency, and job satisfaction was measured. School staff members with equal or less than three completed daily practice logs were set to missing.

#### School actors’ demographics

Regarding school staff’s demographics, experience and familiarity with the local context of a school (tenure) and workload were included as possible confounding variables regarding brokerage or professional well-being. On average, school staff members in our sample had worked for almost 10 years at their present school (*N* = 1,123; *M* = 9.72, *SD* = 9.03). The information on teachers’ percentage of full-time employment (full-time position = 100%) was gathered directly from the principal and recorded with a dichotomous variable (0 = less than 75%, 1 = more than 75%). Staff members employed less than 75% slightly outnumbered the staff employed with at least 75% employment (*N* = 1,297; *M* = 0.44, *SD* = 0.50).

### Data analysis procedures

To answer our research questions, we relied on multiple data analysis procedures: bivariate correlational analysis, measurement invariance analysis, and multiple group structural equation modeling. The different procedures and their contribution to this study are briefly outlined in the following sections.

#### Bivariate correlational analysis

To answer our first research question, we analyzed the associations between the different brokerage aspects (formal, structural, and behavioral) based on bivariate correlational analyses. To answer our second research question, we examined the relationships of the three brokerage facets to the three different aspects of professional well-being (feeling of competency, job satisfaction, and stress) and other teacher demographics (such as tenure and workload) also based on bivariate correlational analysis.

#### Establishing measurement invariance

For our third and last research question, we examined a moderation effect in the differences in brokerage and professional well-being based on (non-) membership in the school’s steering group (formal brokerage). Hence, the question of measurement invariance was addressed in terms of factorial or measurement invariance of the constructs across the two different subgroups (Little et al., 2007). Establishing measurement invariance enabled us to compare the (structural and behavioral) brokerage and professional well-being of school actors who were members or not members of the school’s steering group (formal brokerage). Following Okech (2012), we iteratively conducted analyses to establish configural, weak, and strong invariance.

In a first step, configural invariance was tested in which the same factor structure is maintained across groups. However, as configural invariance only provided a basic and not sufficient requirement for cross-group comparison, further more meaningful degrees of invariance were sought to be established (Little et al., 2007). Hence, in a second step, weak factorial invariance, in which the loadings of the indicators were equated across groups, was tested. However, to compare the constructs meaningfully across groups, measurement equivalence with strong factorial invariance (or at least partial strong factorial invariance) has to be established (Little et al., 2007). Consecutively, in a third step, we fixed both the loadings and the intercepts of the indicators across the groups. In our case, strong invariance means that school actors who participated in steering groups and those who did not will have the same expected scores on the measured indicators of these constructs when they report the same level of (structural and behavioral) brokerage and similar professional well-being. If measurement equivalence is achieved, the examination of similarities and differences in terms of variances, covariances, and means can be analyzed (Okech, 2012). Following the tests of measurement invariance, the relationships were assessed using a multiple group approach to structural equation modeling (SEM).

#### Multiple group analysis

A multiple group SEM (structural equation modeling) technique was applied to test correlations among the constructs across groups. Formal brokerage was used as the grouping variable (0 = no formal brokerage; 1 = formal brokerage).

#### Goodness of fit

Fit indices of the confirmatory factoring analysis, measurement invariance, and multiple group models (such as the RMSEA, CLI, TLI, and SRMR) were estimated by applying a robust maximum likelihood estimator (MLR) for the correction of data that is not normally distributed (Satorra and Bentler, 1994). Additionally, missing data was estimated with the full information maximum likelihood method (Arbuckle et al., 1996). Further, as the assumption of non-independence of the observations was violated, due to a complex nested data structure, we applied a survey design approach—more frequently known as the Type = COMPLEX function in mPlus (Muthen and Satorra, 1995). In this way, unbiased estimators were calculated by introducing the cluster variable ‘school.’

All analyses—confirmatory factoring analysis, bivariate correlational analysis, and multiple group SEM—were computed using the lavaan package Version 0.6-9 (Rosseel, 2012) in R (R Studio-Team, 2020).

## Results

The results are reported in four different subsections, following our research questions and the analytical procedures outlined above. Hence, we first report on the different facets of brokerage and their associations with each other, school actors’ demographics, and professional well-being. To conduct the multiple group analysis for school actors’ being (or not being) formally appointed to act as brokers, we outline the establishment of measurement invariance. Finally, we report the group comparisons in terms of correlation coefficients.

### Bivariate correlational analyses for the different facets of brokerage

To address our first two research questions on the degree of interrelatedness of the different facets of brokerage and their associations with professional well-being we computed bivariate analyses (see Table 2). The model fit was acceptable: χ*^2^* (23) = 98.670, p < 0.001, scaling correction factor Yuan-Bentler correction (Mplus variant) = 0.981: CFI = 0.97, TLI = 0.92; RMSEA [90% CI] = 0.057 [0.046 –0.069], SRMR = 0.021. The covariance matrix indicated that the three brokerage dimensions were only weakly interrelated, revealing that brokerage seems to be indeed a multifaceted concept.

**TABLE 2:** Intercorrelations between demographic variables, brokerage (formal, structural, and behavioral), and school staff’s well-being.

Variables	1	2	3	4	5	6	7
***Demographic variables***							
1. Tenure (in years)	–						
2. Percentage of full-time employment	−0.068*	–					
***Brokerage***							
3. Formal brokerage	*0.053*	0.155***	–				
4. Structural brokerage	0.200***	0.130***	0.273***	–			
5. Behavioral brokerage	*0.025*	*0.045*	0.106*	0.177***	–		
***Well-being***							
6. Competency improving educational practice	0.116**	*–0.023*	0.073**	0.177***	0.270***	–	
7. Satisfaction	0.102**	*−0.008*	*−0.006*	*0.010*	0.065*	0.324***	–
8. Stress	*−0.028*	0.153***	*0.055*	*0.047*	*0.060*	0.095**	–0.313***

Correlation coefficients are given at the individual level. ***p < 0.001, **p < 0.01, *p < 0.05; non-significant effects in italics. Latent construct (5) is based on four items, each rated on a 6-point Likert scale ranging from 1 (strongly disagree) to 6 (strongly agree).

As the significance level for each association among the different facets of brokerage was below the cut-off value of *p* = 0.5, we rejected the null hypotheses and accepted the alternative hypotheses. Hence, our results indicated that formal and structural brokerage were significantly correlated *(hypothesis 1a).* Moreover, in regarding *hypothesis 1b*, formal and behavioral brokerage were also significantly associated with each other. Further, behavioral brokerage also corresponded significantly with structural brokerage (*hypothesis 1c*).

Regarding effect sizes, all the relationships tended to be moderate to weak—with formal to structural brokerage having the strongest association (β = 0.273), followed by the association between structural and behavioral brokerage (β = 0.177). Formal and behavioral brokerage seemed to have the weakest link to each other (β = 0.106).

The results for our second research question indicated that formal (β = 0.055), structural (β = 0.047) and behavioral brokerage (β = 0.060) were not related to school actors’ stress level. Therefore, *hypothesis 2a* could not be accepted. However, an individual’s self-evaluation about feeling competent concerning further developing their school’s educational practice was significantly related to all facets of brokerage (*hypothesis 2b*). The strongest relationship was revealed between behavioral brokerage and school actors reporting feeling competent (β = 0.270). The associations between formal brokerage (β = 0.073) and structural brokerage (β = 0.177) corresponded to a lesser extent with the feeling of competency in further developing the school’s educational practice. Further, school actors in brokerage positions did not report doing and feeling better in terms of job satisfaction—both in terms of formal brokerage (β = –0.006) and structural position in the school’s network (β = 0.010). However, a weak significant correspondence was revealed between brokerage behavior and job satisfaction (β = 0.065). Hence, *hypothesis 2c* could be accepted only partially.

Additionally, regarding demographics, an individual’s workload was significantly related to formal brokerage (β = 0.155) and structural brokerage (β = 0.130). No association was found between behavioral brokerage and school actors’ percentage of full-time employment (β = 0.045). Tenure could only be associated with structural brokerage (β = 0.200). In contrast, both formal brokerage (β = 0.053) and behavioral brokerage (β = 0.025) were not significantly connected with an individual’s seniority at the current school.

### Invariance testing across groups

Establishing measurement invariance across the two groups for our latent construct succeeded only partially (see Table 3a). Based on the model fit indices, configural invariance was supported. Because the first p-value was non-significant, we concluded that weak invariance (equal factor loadings) was supported in this dataset. Hence, the minimal requirements for conducting a multiple group analysis were fulfilled. However, because the second p-value was significant, strong invariance could not be established. Nevertheless, when conducting a multiple group SEM analyses the model revealed viable information, as the fit indices for all the models revealed acceptable values (see Table 3b). This indicated that even when establishing measurement equivalence across the two groups (strong invariance) group comparisons were tenable.

**TABLE 3a:** Comparison of test statistics when establishing configural, weak, and strong measurement invariance across groups.

Model	df	AIC	BIC	χ*^2^*	△χ*^2^*	△df	*P*-value
Configural invariance	28	27,226	27,521	85.395	–	–	–
Weak invariance	31	27,229	27,509	94.132	7.386	3	0.061
Strong invariance	38	27,271	27,517	150.399	59.618	7	< 0.001

**TABLE 3b:** Fit indices for the configural, weak, and strong invariance model.

Model	χ*^2^-Robust*	RMSEA [90% CI]	CFI	TLI	SRMR	Tenable?
Configural invariance	81.794	0.063 [0.047 –0.079]	0.97	0.95	0.024	yes
Weak invariance	89.016	0.063 [0.048 –0.078]	0.97	0.95	0.028	yes
Strong invariance	145.099	0.076 [0.063 –0.089]	0.95	0.92	0.041	yes

### Multiple group analysis

Robust maximum likelihood estimation for the structural equation model based on multiple groups of formal and non-formal brokerage and having considered the nested data structure by introducing the cluster variable ‘school’ revealed a decent model fit: χ*^2^* (28) = 81.794, p < 0.001, scaling correction factor Yuan-Bentler correction (Mplus variant) = 1.044: CFI = 0.97, TLI = 0.95; RMSEA [90% CI] = 0.063 [0.047 –0.079], SRMR = 0.024.

**FIGURE 2:**
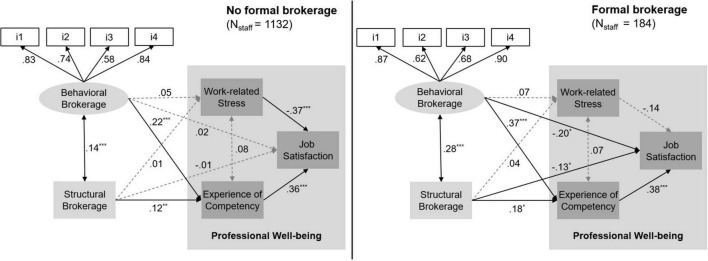
Results of the multiple group structural equation modeling analysis of formal and non-formal brokerage (cluster = school; MLR). χ*^2^* (28) = 81.794, *p* < 0.001, scaling correction factor Yuan-Bentler correction (Mplus variant) = 1.044: CFI = 0.97, TLI = 0.95; RMSEA [90% CI] = 0.062 [0.047 –0.079], SRMR = 0.024. *p < 0.05, **p < 0.01, ***p < 0.001.

Figure 2 shows the path diagrams for the two groups: In broad terms, the direction and significance of the various associations in terms of correlation coefficients did not differ for the two groups. Behavioral brokerage and structural brokerage were both significantly correlated. However, this relationship was much stronger in the formal brokerage group (β = 0.28) than in the no formal brokerage group (β = 0.14). This pattern was also visible when examining the connectedness of behavioral brokerage and competency in further developing educational practice. Whereas interdependence of these aspects was relatively strong in the formal brokerage group (β = 0.37), this association was significantly lower in the other group (β = 0.22). However, there was no significant difference when comparing the associations between structural brokerage and the feeling of being competent in further developing educational practice (formal: β = 0.18; no formal: β = 0.12). In both groups the relationships between brokerage and stress were non-significant. However, a differential effect was revealed when comparing the associations between brokerage and an individual’s job satisfaction: Whereas we identified negative relationships between brokerage and job satisfaction in the formal brokerage group no such effects revealed for the no formal brokerage group. The significant negative associations (more brokerage less satisfied) in the formal brokerage group were stronger for behavioral brokerage (β = –0.20) than for structural brokerage (β = –0.13). However, in the group of actors not formally appointed to fulfill a brokerage position there were no significant relationships between brokerage and job satisfaction—behavioral brokerage (β = 0.02) and structural brokerage (β = –0.01). Hence, when it comes to our third research question *hypothesis 3b* can be accepted. Formal brokers actually being seen as in-betweens by others (structural brokerage) and applying bridge-building strategies (behavioral brokerage) experienced themselves significantly more competent in further developing educational practice when compared to non-formal brokers who were in a brokerage network position and showing brokerage behavior. Moreover, a differential effect in terms of brokerage and job satisfaction (*hypothesis 3c*) revealed. Although in contrast to our hypothesis, formal brokers report to be less and not more satisfied when being in a brokerage position or applying brokerage strategies. No such differential effects were revealed in terms of work-related stress level (*hypothesis 3a*).

## Discussion

The aims of this article were to analyze brokerage in a more comprehensive way and examine its relation to professional well-being. As a first insight, our results reveal that brokerage seems to be indeed a multifaceted concept. A general answer to our first research question therefore is that formal, structural, and behavioral brokerage are different aspects of a school’s collaboration structure. These aspects are interrelated but cannot be treated interchangeably. As a second insight, addressing our second research question, our findings reveal that brokerage and professional well-being correspond only partially. Finally, regarding our third research question, the existence or absence of formal brokerage has only a limited moderating influence on the relationships between individuals’ structural or behavioral brokerage and their professional well-being.

In the following sections we further elaborate on relevant key contributions of this study and present the study’s practical implications. We outline the study’s limitations and where to go next in terms of future research on brokerage. In the concluding section, we sum up the contributions of this study.

### Key contributions and practical implications

Our *first research question* addressed the issue of whether formal, structural, and behavioral brokerage are different approaches to examining a single aspect or multiple aspects of bridge-building interactions among disconnected school actors. As a *first contribution*, our results confirm our assumption that the three facets of brokerage cannot be used interchangeably and that they instead function as complements. However, at least to a certain degree, they correspond to each other. As a first practical implication, we want to indicate that future research in the domain of school improvement needs to describe transparently how they theoretically frame and methodologically assess the brokerage concept.

Going more in-depth, we want to highlight our *second contribution* that formal brokerage is associated with school actors’ brokerage behavior (*hypothesis 1b*). Hence, our results confirm the findings of previous research (i.e., Spillane and Kim, 2012; Daly et al., 2014b). It seems that “title does not dictate behavior” since individuals’ behavioral brokerage is not determined by formal organizational leadership structures. In our case, school staff as members of a steering group only marginally report being more active bridge-builders among disconnected subgroups than school actors having no formal mandate to do so. Therefore, we raise the question as to whether school-internal steering groups actually fulfill their purpose to connect the strategic center of the school (leadership group) to the periphery (i.e., subgroups). There are multiple tentative explanations for these findings.

For instance, formal brokers may lack instructions concerning the aims of a steering group and therefore may simply not be aware of the importance of applying brokerage behavior, acting as gatekeepers and representatives. As a second practical implication, then, school actors may need to critically reflect on the purpose and manifestation of formal leadership groups in their organization.

Our second interpretation is related to the “autonomy parity pattern” (Lortie, 1975) among teachers that is at stake when implementing a middle leadership structure such as a steering group (Feldhoff et al., 2010). Members of a steering group may be confronted with the rejection by other staff members—which may result first in constraining their potential to build bridges and in the long run may inhibit their willingness to be active in-betweens. Again, school staff members need to have the opportunity to express their doubts and address their concerns when new leadership structures are implemented. This might be done in collective sense-making processes (Coburn, 2005) in which means and ends of formalized brokerage structures are presented, discussed, and, if necessary, adjusted for the local context. In these negotiation processes, school teams, for instance, can develop a more differentiated understanding of what teacher autonomy is and come to see that more cooperative practice is not necessarily a threat to an individual’s autonomy (Vangrieken et al., 2017).

Finally, our third interpretation addresses the issue that social bridges in a school’s network are built to a great extent in informal ways. From a socio-constructivist perspective, this finding mirrors the fact that schools are more organic than mechanical organizations (Mitchell and Sackney, 2011). Hence, school actors not officially mandated to act as brokers may sidetrack the formal organizational structure (Spillane and Kim, 2012).

There is more evidence for such a sidetracking effect of formal leadership structures by informal brokers: As correspondence of an individual’s formal to structural brokerage (*hypothesis 1a*) is only moderately strong, the steering group members’ structural brokerage position does not necessarily reflect the formal leadership structure. This is the *third contribution* of our study: Confirming previous studies about brokerage in the domain of school improvement (i.e., Spillane and Kim, 2012; Daly et al., 2014a) network structures are influenced but not determined by formal organizational structures.

Our *fourth and final contribution* related to the first research question is that, as *a priori* hypothesized, structural and behavioral brokerage are interrelated (*hypothesis 1c*)—although again merely to a modest degree. Hence, actors in a brokerage network position do tend to apply brokerage activities more often. However, there is room for actors not in a brokerage position to fulfill the role of linking agents, or for individuals that are identified as brokers from a structural perspective to refrain from taking advantage of their position. At this point, we can only speculate whether they cannot or do not want to apply brokerage behavior, or whether they are simply not aware of the fact that they are intermediaries between different subgroups and individuals within the school’s social network.

Our *second research question* was about the relationships between the different facets of brokerage and professional well-being. As a *fifth contribution*, our findings do not confirm that brokerage is related to more work-related stress. This is in contrast to previous studies outside of the school context that found that individuals building bridges among disconnected others more often report a higher stress level (i.e., Krackhardt, 1999; Mollenhorst et al., 2015; Kislov et al., 2017). Hence, we had to reject our *hypothesis 2a*.—neither formal, structural or behavioral brokerage are reflected in higher stress. Thus, when referring to Deci and Ryan’s (2008) work about well-being, school staff members’ brokerage seems not to be related to presence or absence of negative affect (*hedonic aspect* of professional well-being).

However, there is a different picture evolving when it comes to an individual’s experience of competency in further developing the school’s educational practice (*eudaimonic aspect* of school staff members’ professional well-being). We assumed that brokerage is more positively associated with an individual’s experience of competency when it comes to further developing their school’s educational practice (*hypothesis 2b*). As a *sixth contribution* we want to emphasize that our results confirm this relationship—which up to now had been only examined based on small samples in qualitative case studies (e.g., Slavit and Roth McDuffie, 2013; Jusinski, 2019). In addition, our results reveal a more fine-grained picture: Whereas brokerage behavior is substantially associated with the experience of competency, this association is weaker when it comes to structural brokerage. Being in a formal brokerage position, however, is only marginally correlated with this aspect of professional well-being. As a practical implication derived from these findings, we suggest making school staff members in formal brokerage positions more aware of the importance of brokerage behavior. Moreover, formal brokers need to be well-equipped with strategies for transferring knowledge, linking disconnected others, or supporting capacity-building processes (Ward et al., 2009).

Concerning job satisfaction, we examined whether there is a positive relationship between brokerage and the tendency to not only do but also feel better (*hypothesis 2c*). The *seventh contribution* of this paper is that there is no such association when it comes to formal and structural brokerage in the school context. However, brokerage behavior is weakly interconnected with job satisfaction. These findings indicate that the tendency to feel better is only related to actual brokerage behavior. There are multiple reasons why this evidence, most prominently advocated by Burt (2005), does not hold true for school staff members: Possible explanations may be, the less hierarchical leadership structures, the limited monetary incentives available to reward staff members, or the limited career perspectives in most primary schools (Rowan, 1994; Ingersoll and Collins, 2018). Although educators act as brokers within their schools, their brokerage seems to be not that rewarding. This is critical, as previous research indicated that brokerage might be beneficial when it comes to changing organizational practice sustainably. Hence, educational policymakers and central district staff should come up with ideas and solutions on how to create conditions in which school actors can profit individually when taking up formal or structural brokerage positions.

Finally, our *third and final research question* examined a potential moderation effect in terms of formal brokerage. We followed the argumentation of Hopkins et al. (2013) and Nordholm (2016) that a lack of formal legitimacy diminishes the benefits related to an individual’s brokerage. Hence, we assumed that a school staff member’s formal mandate to act as a gatekeeper or representative of subgroups within the school organization is more significantly associated with professional well-being—in terms of a lower stress level (*hypothesis 3a*), a more positive experience of competency (*hypothesis 3b*) and a higher level of job satisfaction (*hypothesis 3c*). As an *eight contribution*, a general picture of our results indicates that the two groups of no formal brokerage and formal brokerage do not differ substantially when it comes to the interrelatedness of structural and behavioral brokerage and also when it comes to their associations with work-related stress. Thus, both formal and no formal brokers do not report to be experience a higher stress level when compared to their colleagues.

However, *our ninth contribution* in the form of a more fine-grained analysis of the two groups reveals differential effects in terms of the strength and directions of some correlations. In both groups, formal and no formal brokers, brokerage behavior is associated with the feeling of competency. However, this relationship is significantly more intense in the group of formal brokers. As a practical implication, therefore, we suggest either thinking about formalizing brokerage within the school or to making sure that in the long run informal brokerage is made more visible, appreciated, and rewarded. Our findings reveal another difference considering job satisfaction. Whereas both structural and behavioral brokerage in the non-formal group is not related to job satisfaction our results show that in the formal brokerage group these relationships are significant. However, in contrast to our hypothesis, these effects are negative. This last differential effect is not in line with previous studies (Hopkins et al., 2013; Nordholm, 2016). Having a formal mandate to build bridges in this regard is not more but less rewarding on an individual level. These mixed results in terms of formal legitimacy indicate that there is no single best solution. Whereas formalizing brokerage diminishes an individual’s job satisfaction, it increases the experience of competency. Hence, it might be about weighing the benefits and drawbacks carefully when implementing new formal leadership structures.

### Limitations and further research

The study has several limitations that should be noted: As a first limitation of this study, we want to acknowledge that this study was only exploratory in its nature. The bivariate correlational analyses applied offer a first insight about how the different facets of brokerage interrelate and how school staff members’ brokerage is associated with their professional well-being. However, social network data is dependent data (Robins, 2015). School staff members’ betweenness-centrality (structural brokerage) is therefore a relational information and depending on the positions of all their other colleagues in the school’s professional network. Thus, the assumption that all variables in our statistical models were observed independently was violated (Hox et al., 2017). Future research needs to validate the associations of brokerage with school staff members’ professional well-being based on more sophisticated methodologies that combine multigroup SEM with social network analyses. For instance, the multiple-membership multiple-classification models for social network and group dependencies by Tranmer et al. (2014) might provide a promising starting point to bring these two methodologies together.

A second limitation is that we did not collect data about a school’s history with its steering group. Future studies might take a closer look at for how long a formal leadership group has been in place, or whether there were any more or less successful episodes related to the leadership group.

Another limitation of the study is that the way formal brokers were selected from the school team has been neglected. There are different reasons why certain school actors join a formal leadership group. Some school actors might be highly motivated and willing to join the group voluntarily. Others are less committed and see this position merely as a compulsory task. These differences might be essential when examining an individuals’ brokerage position, brokerage behavior, and professional well-being.

Moreover, our study relied on cross-sectional data only. Future studies with longitudinal designs might further examine the relationships between brokerage and professional well-being—also in causal directions.

Finally, there has been criticism of assessing individuals’ brokerage based on the betweenness-centrality. For example, Gould and Fernandez (1989) argued that solely being multiple times on long paths from one end of the network to the other does not necessarily indicate “a very important role in purposive social interaction” (p. 95). Keeping track of and making use of the complex social interaction patterns several steps beyond the people to whom an individual is directly connected seems impractical. Gould and Fernandez therefore suggested focusing exclusively on the direct links connecting otherwise disconnected actors. Hence, scholars may conduct brokerage research based on alternative approaches when assessing an individual’s structural position.

### Conclusion

In this study, we wanted to raise the question of whether school actors being formally entitled to act as intermediaries between the school principal and the various sub-teams within their schools actually demonstrate bridge-building behavior. Or to what degree school actors lacking formal entitlement still act as gatekeepers and representatives – just in informal ways. Our study revealed on an exploratory basis that school actors’ entitlement to act as formal brokers does not dictate their behavior. However, the degree individuals take up a brokerage position in the school’s social network and their bridge-building behavior are both influenced by formal brokerage. Hence, in this case, title does not determine but affects an individual’s behavior.

Moreover, we addressed the research question whether brokerage is related to professional well-being in a multifaceted way. We conclude that our findings contribute to a better understanding of the social capital of school staff within a school organization and also to the exploration of how social positions are related to important psychological dimensions. To sum up, our results point out that brokerage and professional well-being have to be assessed in a context-sensitive way. The positive relationships that have been found between having an intermediary position and professional well-being seem to be true only when it comes to eudaimonic aspects of professional well-being (in our case, an individual’s experience of competency when further developing educational practice). No such effects were identified in terms of hedonic aspects of professional well-being (work-related stress). However, in contrast to previous findings, our study revealed that building bridges in a formal brokerage position is negatively related to job satisfaction. These findings emphasize the urgent need to address the issue of professional well-being in a differentiated way when it comes to the context of school improvement. The brokerage concept is just one way to start.

## Data Availability Statement

The raw data supporting the conclusions of this article will be made available by the authors as soon as the research project is finished, without undue reservation. Requests to access the datasets should be directed to BR, beat.rechsteiner@ife.uzh.ch.

## Ethics statement

The studies involving human participants were reviewed and approved by Ethikkomission der Philosophischen Fakultät (University of Zurich). The patients/participants provided their written informed consent to participate in this study.

## Author contributions

KMM, AW, and BR organized the database. BR wrote the first draft of the manuscript with input from all authors. BR and KMM developed the theoretical assumptions in the introduction. BR wrote the section about Materials and Methods and performed the statistical analyses. MC, AW, and KMM verified the analytical methods. KMM and AW supervised the research project. All authors discussed the results and contributed to the final manuscript, conception and design of this study.

## Conflict of Interest

The authors declare that the research was conducted in the absence of any commercial or financial relationships that could be construed as a potential conflict of interest.

## Publisher’s Note

All claims expressed in this article are solely those of the authors and do not necessarily represent those of their affiliated organizations, or those of the publisher, the editors and the reviewers. Any product that may be evaluated in this article, or claim that may be made by its manufacturer, is not guaranteed or endorsed by the publisher.
